# Radical-Scavenging Activity and Ferric Reducing Ability of* Juniperus thurifera* (L.),* J. oxycedrus* (L.),* J. phoenicea* (L.) and* Tetraclinis articulata* (L.)

**DOI:** 10.1155/2016/6392656

**Published:** 2016-05-16

**Authors:** Meryem El Jemli, Rabie Kamal, Ilias Marmouzi, Asmae Zerrouki, Yahia Cherrah, Katim Alaoui

**Affiliations:** ^1^Pharmacodynamy Research Team, ERP, Laboratory of Pharmacology and Toxicology, Faculty of Medicine and Pharmacy, Mohammed V University, BP 6203, Rabat-Instituts, Rabat, Morocco; ^2^Laboratory of immunology, Faculty of Medicine and Pharmacy, Mohammed V University, Rabat, Morocco

## Abstract

*Objective*. The aim of this work is to study and compare the antioxidant properties and phenolic contents of aqueous leaf extracts of* Juniperus thurifera*,* Juniperus oxycedrus*,* Juniperus Phoenicea,* and* Tetraclinis articulata* from Morocco.* Methods*. Antioxidant activities of the extracts were evaluated by 2,2-diphenyl-1-picrylhydrazyl (DPPH) free radical-scavenging ability, Trolox equivalent antioxidant capacity (TEAC), and ferric reducing antioxidant power (FRAP) assays. Also the total phenolic and flavonoids contents of the extracts were determined spectrophotometrically.* Results*. All the extracts showed interesting antioxidant activities compared to the standard antioxidants (butylated hydroxytoluene (BHT), quercetin, and Trolox). The aqueous extract of* Juniperus oxycedrus *showed the highest antioxidant activity as measured by DPPH, TEAC, and FRAP assays with IC_50_ values of 17.91 ± 0.37 *μ*g/mL, 19.80 ± 0.55 *μ*g/mL, and 24.23 ± 0.07 *μ*g/mL, respectively. The strong correlation observed between antioxidant capacities and their total phenolic contents indicated that phenolic compounds were a major contributor to antioxidant properties of these plants extracts.* Conclusion*. These results suggest that the aqueous extracts of* Juniperus thurifera*,* Juniperus oxycedrus*,* Juniperus phoenicea,* and* Tetraclinis articulata* can constitute a promising new source of natural compounds with antioxidants ability.

## 1. Introduction 

Researchers are now looking for natural antioxidants which do not have any side effects on human health. The search is underway to find out newer, effective, and safe antioxidants, in order to use them in foods and pharmaceutical preparations to replace the synthetic ones.

Medicinal plants are the major source of chemical compounds exhibiting antioxidant activity. Several studies have reported the interesting composition of Moroccan medicinal plants, including phenolic acids, flavonoids, and tannins, which are known for their health benefits as antioxidants [1–3].

The Moroccan flora contains more than 4200 vascular plant species. However, few ones were screened for pharmacological or chemical properties [4]. Among them, the Cupressaceae is the most common family of conifers (gymnospermae) throughout the world. In fact, they make a striking impression on the plant landscape of the mediterranean basin and are one of world's biodiversity hotspots. The Cupressaceae grows up from the coast up to high altitudes, also able to develop in extreme ecological conditions and considered among the most important aromatic plants in the Moroccan traditional medicine.

Recent investigation has reaffirmed the validity of many of their traditional uses and reported that the extracts from Cupressaceae contain most of the important phenolic compounds, especially flavonoids, neolignans, and phenylpropanoids [5–7]. In this context and based on taxonomic criteria indicating close similarities between the four chosen species (*Juniperus thurifera*,* Juniperus phoenicea*,* Juniperus oxycedrus,* and* Tetraclinis articulata*), it is interesting to evaluate the chemotaxonomic and pharmacological differences characterizing this plant family. These species have many uses in traditional medicine in several parts of the world.* Juniper* berries are used as a spice, particularly in European cuisine, which are the only spice derived from conifers. In Morocco, Cupressaceae tar, leaves, and fruits are widely used to treat different hair and skin problems like dandruff, eczema, itchiness, and fungal infections [8]. Additionally, infusions of Cupressaceae species of dried leaves are used internally to treat rheumatism, diarrhea, and diabetes mellitus. These health benefits could be in part attributed to the potential effects of their antioxidants such as phenolic compounds on the reactive oxygen species produced in the human body [9, 10].

Therefore, the present study aims to determine and compare the antioxidant potential of aqueous leaf extracts of* Juniperus thurifera*,* Juniperus phoenicea*,* Juniperus oxycedrus,* and* Tetraclinis articulata* using DPPH free radical-scavenging activity (DPPH), Trolox equivalent antioxidant capacity (TEAC), and ferric reducing antioxidant power (FRAP) assays and moreover to determine their total phenolic contents and investigate the relationship between total phenolic content and antioxidant activity.

## 2. Material and Methods 

### 2.1. Plant Materials

In February 2014, leaves of* J. thurifera*,* J. Phoenicea*,* J. oxycedrus,* and* T. articulata* were collected from wild populations located in Oukaimeden, Essaouira, Ourika, and Ait Issi Ihahan regions, respectively. The characteristics of collection sites are shown in Table 1. The leaves were identified by Pr. Fennane, and vouchers specimens were deposited at the herbarium of the Scientific Institute of Rabat and referred to as 79594 (*J. thurifera*), 79592 (*J. phoenicea*), 79588 (*J. oxycedrus*), and 79589 (*T. articulata*). The collected plant material was air-dried at room temperature (20–24°C). Leaves of dried plant were separated from plant and stored in tight-seal dark containers until use.

### 2.2. Preparation of Plant Extracts

To prepare the water extract, 10 g of dry powdered plant material was soaked in 100 mL of boiling distilled water and left to stand at room temperature for 30 min. The hot infusions were then filtered using Whatman filter paper and concentrated under vacuum on a rotary evaporator at 60°C and stored at 4°C for further use.

### 2.3. Antioxidant Activity

#### 2.3.1. DPPH Free Radical-Scavenging Activity

The ability of water extracts of* J. thurifera*,* J. Phoenicea*,* J. oxycedrus,* and* T. articulata* to scavenge the DPPH radical was estimated using the method described by Şahin et al. [11]. An aliquot of 50 *μ*L of various sample concentrations was added to a volume of 2 mL from the DPPH methanolic solution (60 *μ*M). The reaction mixture was well shaken and incubated for 20 min at room temperature in the dark and the absorbance was recorded at 517 nm. The blank was constituted by methanol instead of the extract. Butylated hydroxytoluene (BHT) and quercetin were used as positive controls. The percentage inhibition of the DPPH radical by the samples was calculated using the following equation:

(1) $$\begin{array}{c} \%\text{ inhibition}=\frac{\left(A_{0}-A_{1}\right)}{A_{0}}\times 100, \end{array}$$

where *A*_0_ is the absorbance of control sample and *A*_1_ is the absorbance of the test sample. The sample concentration providing 50% of inhibition (IC_50_) was determined from the plotted curve of inhibition using several concentrations.

#### 2.3.2. Trolox Equivalent Antioxidant Capacity (TEAC) Assay

The ABTS free radical-scavenging activity of each sample was determined according to the method described by Loizzo et al. [12]. The ABTS radical cation was produced by reacting ABTS with potassium per sulfate. A mixture of ABTS (2 mM) and potassium persulfate (70 mM) was allowed to stand overnight at room temperature in the dark to form the radical cation ABTS, 16 h prior to use. The ABTS solution was then diluted with 80% methanol to obtain an absorbance of 0.700 ± 0.005 at 734 nm. 100 *μ*L of appropriately diluted samples was added to 2 mL of ABTS solution and the absorbance was recorded at 734 nm after 1 min of incubation at room temperature. A standard curve was obtained by using Trolox standard solution at various concentrations (ranging from 0 to 0.24 *μ*g/mL). The scavenging activity of different concentrations of extracts and fractions against ABTS radical were also measured to calculate IC_50_, and the procedure was similar to the DPPH scavenging method described above.

#### 2.3.3. Reducing Power Determination

The ferric reducing capacity of extracts was investigated by using the potassium ferricyanide-ferric chloride method [13]. Briefly, 0.2 mL of each of the extracts at different concentrations, 2.5 mL of phosphate buffer (0.2 M, pH 6.6), and 2.5 mL of potassium ferricyanide K_3_Fe(CN)_6_ (1%) were mixed and incubated at 50°C for 20 min, to reduce ferricyanide into ferrocyanide. The reaction was stopped by adding 2.5 mL of 10% (w/v) trichloroacetic acid followed by centrifugation at 1000 rpm for 10 min. Finally, 2.5 mL of the upper layer was mixed with 2.5 mL of distilled water and 0.5 mL of FeCl_3_ (0.1%) and the absorbance was measured at 700 nm. The sample concentration providing 0.5 of absorbance (IC_50_) was calculated by plotting absorbance against the corresponding sample concentration. BHT and quercetin were used as a reference compound.

#### 2.3.4. Determination of Total Phenolic Content

The total phenolic contents (TPC) were determined spectrophotometrically according to the Folin-Ciocalteu colorimetric method [14]. Briefly, 20 *μ*L aliquot of extract solution was mixed with 1.16 mL of distilled water and 100 *μ*L of Folin-Ciocalteu reagent, followed by the addition of 300 *μ*L of Na_2_CO_3_ solution (20%). After 30 min of incubation at 40°C, the absorbance of the reaction mixture was measured at 760 nm. Gallic acid was used as a reference standard, and the results were expressed as microgram gallic acid equivalent (*μ*g GAE)/mg dry weight of plant extract (edw).

#### 2.3.5. Determination of Total Flavonoid Content

Total flavonoid content was measured by the aluminum chloride colorimetric assay [15]. 0.5 mL of the extracts or standard solution of quercetin was mixed with 0.5 mL of 2% AlCl_3_. After 1 hour at room temperature, the absorbance was measured at 420 nm. Total flavonoid content was expressed as microgram quercetin equivalent (*μ*g QE)/mg dry plant extract (edw).

### 2.4. Statistical Analysis

All the assays were carried out in triplicate. The means and standard deviation (SD) were determined using SPSS version 20, the results of antioxidant activities and phenolic compounds composition are expressed as mean values ± SD, and the analysis of variance was performed to determine significant differences.

## 3. Results 

### 3.1. Antioxidant Activities

Antioxidant activity is a complex procedure usually happening through several mechanisms and is influenced by many factors, which cannot be fully described with one single method. Therefore, it is essential to perform more than one type of antioxidant capacity measurement to take into account the various mechanisms of antioxidant action [16–18]. In this study, three complementary tests were used to assess the antioxidant activity of* J. thurifera*,* J. phoenicea*,* J. oxycedrus,* and* T. articulata* infusions: DPPH free radical-scavenging activity, Trolox equivalent antioxidant capacity (TEAC), and reducing power assays.

As summarized in Table 2, the rank order of antioxidant potency was the same for all assays, namely, in decreasing order,* J. oxycedrus*, followed by* J. thurifera*,* T. articulata,* and* J. oxycedrus*.

All extracts were able to reduce the stable, purple-coloured radical DPPH into yellow-coloured DPPH-H. The water extract obtained from* J. oxycedrus* had the strongest free radical-scavenging activity with IC_50_ value of 17.91 ± 0.37 *μ*g/mL (Table 2). On the other hand the lowest capacity to reduce DPPH was observed in* J. phoenicea* water extract (IC_50_ = 30.74 ± 0.11 *μ*g/mL).

All water extracts were less effective than the synthetic antioxidant BHT (IC_50_ = 4.20 ± 0.02 *μ*g/mL) and quercetin (IC_50_ = 1.29 ± 0.01 *μ*g/mL). Similarly,* J. oxycedrus* extract exhibited the best performance in ABTS and reducing power assays with IC_50_ = 19.80 ± 0.55 *μ*g/mL and 24.23 ± 0.07 *μ*g/mL, respectively.

### 3.2. Total Phenolic Compounds

**Figure 1:**
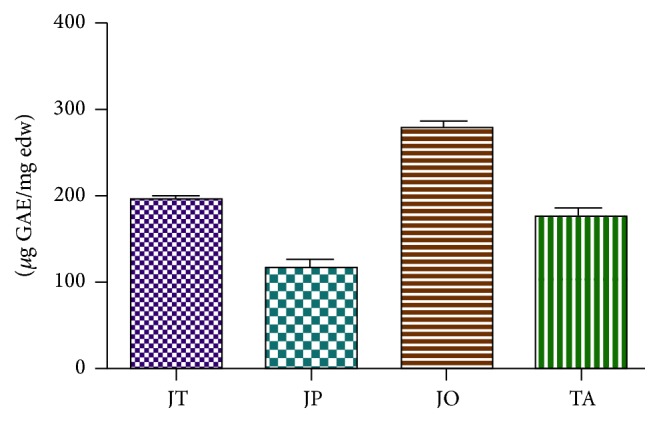
Total polyphenol content expressed as gallic acid equivalents (*μ*g GAE)/mg plant extract in* J. thurifera* (JT),* J. oxycedrus* (JO),* J. phoenicea* (JP), and* T. articulata* (TA) infusions. Data are expressed as mean ± SD (*n* = 3).

The aqueous extracts of* J. thurifera*,* J. phoenicea*,* J. oxycedrus,* and* T. articulata* were characterized by the presence of considerable amount of phenolic compounds (Figure 1). The highest amount of total phenolics was found in* J. oxycedrus* with (278.56 ± 9.67) *μ*g GAE/mg edw, followed by* J. thurifera* (193.79 ± 6.47) *μ*g GAE/mg edw,* T. articulata* (175.67 ± 10.21) *μ*g GAE/mg edw, and* J. phoenicea* (116.35 ± 9.71) *μ*g GAE/mg edw.

### 3.3. Total Flavonoid Content

**Figure 2:**
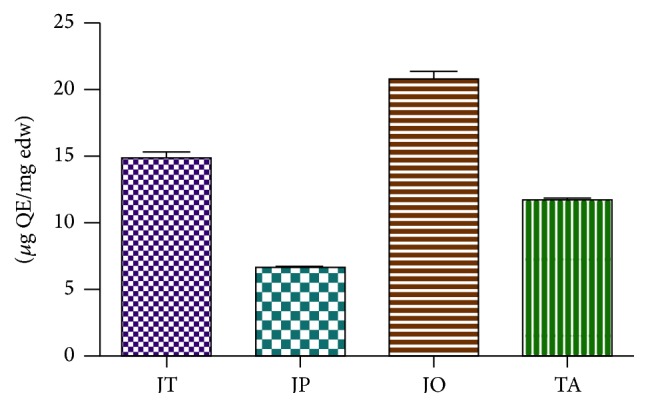
Total flavonoid content expressed as quercetin equivalents (*μ*g QE)/mg plant extract in* J. thurifera* (JT),* J. oxycedrus* (JO),* J. phoenicea* (JP), and* T. articulata* (TA) infusions. Data are expressed as mean ± SD (*n* = 3).

Concentration of flavonoids in investigated species ranged from 6.70 to 20.81 *μ*g (QE)/mg edw (Figure 2). A high concentration of flavonoids was determined in* J. oxycedrus* accounting for 20.81 ± 0.63, 14.93 ± 0.40, and 11.78 ± 0.30 *μ*g (QE)/mg edw in* J. thurifera* and* T. articulata,* respectively. The lowest flavonoid concentration was determined for* J. phoenicea* (6.69 ± 0.22 *μ*g (QE)/mg edw).

### 3.4. Relationship between Total Antioxidant Capacity and Phenolic Content

To evaluate the suitability and reliability of the three assay methods used to determine the total antioxidant capacities of the four Cupressaceae species, we performed correlation analysis of the values of total antioxidant capacity obtained by these methods. As shown in Table 3, all *R* ^2^ values were positive at *P* < 0.01 significance level, indicating that the values of antioxidant capacities assayed by the three different methods were highly correlative. DPPH values were strongly correlated with the ABTS activities (*R* ^2^ = 0.951) and reasonably well correlated with the FRAP values (*R* ^2^ = 0.957). The correlation between the ABTS and FRAP values was also highly significant (*R* ^2^ = 0.848).

### 3.5. Correlation between Total Phenolic Content, Flavonoid Content, and Antioxidant Activity

The correlation between antioxidant capacity and phenolic content of the four Moroccan Cupressaceae samples is described in Table 3. The antioxidant activity data obtained from the DPPH method were highly correlated with the total phenolic contents (*R* ^2^ = 0.986) and total flavonoids concentration (*R* ^2^ = 0.986). The total phenolic and flavonoid content also correlated well with the ABTS (*R* ^2^ = 0.967; *R* ^2^ = 0.981) and FRAP values (*R* ^2^ = 0.911; *R* ^2^ = 0.929).

## 4. Discussion 

Oxidative stress has been implicated in several diseases including diabetes, rheumatoid arthritis, cardiovascular diseases, atherosclerosis, neurodegenerative diseases (Parkinson, Alzheimer, and Huntington), cancer, and aging [19–21]. Natural antioxidants such as phenolic acids and flavonoid compounds from plants may offer resistance against the oxidative stress by scavenging free radicals, inhibiting lipid peroxidation, and by other mechanisms [22, 23]. Thus the present study was undertaken with the aim to show the antioxidant potentials of four Cupressaceae species known to be used in folk medicine in Morocco using three widely known methods (DPPH, ABTS, and FRAP) and also to establish the possible correlations between the antioxidant activity and total phenolic and flavonoid contents of the extracts.

In fact the used methods have different reaction mechanisms [24]. For instance, DPPH and ABTS assays are based on electron and H atom transfer, while the FRAP assay is based on electron transfer reaction [24–26]. However, the three methods clearly indicated that the studied plants possess considerable antioxidant and antiradical activities.

Furthermore, well-pronounced correlations were observed between these methods which confirm that the three assays were all suitable and reliable for assessing total antioxidant capacities of the plant extracts.

The results of our screening on the four Cupressaceae species growing in Morocco showed that the leaf water extracts of* J. oxycedrus* have the strongest scavenging activity against DPPH (17.91 ± 0.37 *μ*g/mL), ABTS (19.80 ± 0.55 *μ*g/mL), and the best performance reducing power activity (24.23 ± 0.07 *μ*g/mL) followed by the leaf extract of* J. thurifera* (24.85 ± 0.42 *μ*g/mL (DPPH); 30.36 ± 0.24 *μ*g/mL (ABTS); 35.83 ± 0.37 *μ*g/mL (FRAP)),* T. articulata* (27.38 ± 0.02 *μ*g/mL (DPPH); 32.92 ± 0.56 *μ*g/mL (ABTS); 47.12 ± 0.15 *μ*g/mL (FRAP)), and* J. phoenicea* (30.74 ± 0.11 *μ*g/mL (DPPH); 47.37 ± 0.59 *μ*g/mL (ABTS); 46.85 ± 0.42 *μ*g/mL (FRAP)) (Table 2). This work also showed that the main polyphenolic class (flavonoid and phenolic acids) was common among the different species and some polyphenolic compounds have been identified previously in various studies for different Cupressaceae species [27–30].

There have been diverse reports on antioxidant activity of a number of Cupressaceae species. The leaves and berries of* J. phoenicea* showing a strong antioxidant activity in ABTS and DPPH radical-scavenging activity test afforded several phenolics (tannins, anthocyanins, and flavonoids), which were stated to be the free radical scavengers in this plant [31]. In a study on antioxidant activity of the Turkish* Juniperus* [32], the aqueous and ethanolic extracts of the fruits and leaves from* J. oxycedrus* were examined using two different tests of the ferric reducing antioxidant power assay and DPPH radical-scavenging activity and most of them showed a potent antioxidant effect under those systems, which is in accordance with our data. High DPPH scavenging activity of* Tetraclinis articulata* leaves collected in Tunisia was also reported [33].

The strong correlations between the total antioxidant capacity assayed by DPPH, ABTS, and FRAP methods and the phenolic content (Table 3) indicate that the phenolic compounds largely contribute to the antioxidant activities of these Cupressaceae species and therefore could play an important role in the beneficial effects of these important medicinal plants. The results were in accordance with other researches, several studies have found that phenolic compounds are major antioxidant constituents in selected plants, and there are direct relationships between their antioxidant activity and total phenolic content [34–37].

## 5. Conclusion 

The findings obtained in this study support the traditional uses of those plant species as therapeutic agents. The higher antioxidant potential of those Moroccan Cupressaceae is conferred by their high phenolic content.* J*.* oxycedrus* presented the highest TPC, TFC, and antioxidant capacity values. In addition, there was a good correlation between phenolic content and antioxidant capacity of the leaves extracts. These results suggest that the water infusion from the leaves of* J. thurifera*,* J. oxycedrus*,* J. phoenicea,* and* T. articulata* constitutes a valuable source of antioxidant metabolites. Further investigations are required to identify their active metabolites.

## Figures and Tables

**Table 1:** Collection site and geographical coordinates of the species studied and percentage yields (%) of the water extract obtained.

Species	Collection site	Voucher specimens	Latitude/longitude	Altitude (m)	Yield % (v/w)
JT	Oukaimeden	79594	N 31°14′/W 07°41′	2700	17.69 ± 0.22
JP	Essaouira	79592	N 31°10′/W 09°30′	724	20.13 ± 0.05
JO	Ourika	79588	N 31°13′/W 08°03′	1170	18.01 ± 0.08
TA	Ait Issi Ihahan	79589	N 30°91′/W 09°43′	1300	16.87 ± 0.38

**Table 2:** IC_50_ values (*µ*g/mL) of *J. thurifera, J. oxycedrus, J. phoenicea, *and* T. articulata* infusions and of BHT, quercetin, and Trolox.

Assays	Infusions				Positive control		
	*J. thurifera*	*J. phoenicea*	*J. oxycedrus*	*T. articulata*	BHT	Quercetin	Trolox
DPPH	24.85 ± 0.42	30.74 ± 0.11	17.91 ± 0.37	27.38 ± 0.02	4.20 ± 0.02	1.29 ± 0.01	—
ABTS	30.36 ± 0.24	47.37 ± 0.59	19.80 ± 0.55	32.92 ± 0.56	—	—	1.93 ± 0.01
FRAP	35.83 ± 0.37	46.85 ± 0.42	24.23 ± 0.07	47.12 ± 0.15	7.02 ± 0.02	2.06 ± 0.01	—

Values represent means ± SD (standard deviations) for triplicate experiments.

**Table 3:** Correlation coefficient among antioxidant assays and total phenolic and flavonoid contents.

	DPPH	ABTS	FRAP
ABTS	0.951	—	—
FRAP	0.957	0.848	—
TPC	0.986	0.967	0.911
TFC	0.986	0.981	0.929

Correlation is significant at *P* < 0.01.

## Abbreviations

DPPH: Free radical-scavenging activity
TEAC: Trolox equivalent antioxidant capacity
FRAP: Ferric reducing antioxidant power
BHT: Butylated hydroxytoluene
*J. thurifera*: * Juniperus thurifera*
*J. phoenicea*: * Juniperus phoenicea*
*J. oxycedrus*: * Juniperus oxycedrus*
*T. articulata*: * Tetraclinis articulata*
IC_50_: Concentration providing 50% inhibition
TPC: Total phenolic contents
TFC: Total flavonoid content
SD: Standard deviation.
